# EF-Hand-Binding Secreted Protein Hdh-SMP5 Regulates Shell Biomineralization and Responses to Stress in Pacific Abalone, *Haliotis discus hannai*

**DOI:** 10.3390/cimb45120629

**Published:** 2023-12-13

**Authors:** Md Abu Hanif, Ji Do Han, Soo Cheol Kim, Shaharior Hossen, Kang Hee Kho

**Affiliations:** 1 Department of Fisheries Science, Chonnam National University, Yeosu 59626, Republic of Korea; hanif@jnu.ac.kr (M.A.H.); shaharior@jnu.ac.kr (S.H.); 2 South Sea Fisheries Research Institute, National Institute of Fisheries Science, Yeosu 59780, Republic of Korea; onemap83@gmail.com (J.D.H.); sckim9194@korea.kr (S.C.K.)

**Keywords:** Pacific abalone, secreted protein, EF hand, biomineralization, starvation, thermal stress

## Abstract

The development of a shell is a complex calcium metabolic process involving shell matrix proteins (SMPs). In this study, we describe the isolation, characterization, and expression of SMP5 and investigate its potential regulatory role in the shell biomineralization of Pacific abalone *Haliotis discus hannai*. The full-length *Hdh-SMP5* cDNA contains 685 bp and encodes a polypeptide of 134 amino acids. Structurally, the Hdh-SMP5 protein belongs to the EF-hand-binding superfamily, which possesses three EF-hand Ca^2+^-binding regions and is rich in aspartic acid. The distinct clustering patterns in the phylogenetic tree indicate that the amino acid composition and structure of this protein may vary among different SMPs. During early development, significantly higher expression was observed in the trochophore and veliger stages. *Hdh-SMP5* was also upregulated during shell biomineralization in Pacific abalone. Long periods of starvation cause *Hdh-SMP5* expression to decrease. Furthermore, *Hdh-SMP5* expression was observed to be significantly higher under thermal stress at temperatures of 15, 30, and 25 °C for durations of 6 h, 12 h, and 48 h, respectively. Our study is the first to characterize Hdh-SMP5 comprehensively and analyze its expression to elucidate its dynamic roles in ontogenetic development, shell biomineralization, and the response to starvation and thermal stress.

## 1. Introduction

Shells represent the mastery of cellular-engineered microstructures, which form common features of the phylum Mollusk and are emblematic of the morphological diversity in nature [1]. This biomineralized tissue performs a diverse array of functions, such as the storage of mineral ions, tissue support, protection of the soft body from predators, and, to some extent, from environmental factors [2]. Most mollusks possess a single, helically coiled shell over their entire lifetime, which grows as they increase the number of whorls [3]. Typically, this shell exhibits a three-layered structure, with two inner calcified layers (prismatic and nacreous) and an outermost organic thin layer (periostracum) [4]. The prismatic layer is composed of prism-shaped calcific crystals, and the nacreous layer is made up of aragonite crystals, which form a brick wall-like structure. This natural biomaterial comprises mainly CaCO_3_ and cell-free organic macromolecules (proteins, lipids, carbohydrates, glycoproteins, polysaccharides, peptides, and chitins), which are secreted by the outer mantle epithelial cells [5,6]. Generally, the shell of mollusks is made of CaCO_3_ (95%) and organic matrix (1–5%) [7].

Although the genesis, physical substances, and patterning of the molluscan shell have been the attention of researchers and scientists for several decades, the molecular and biochemical mechanisms underlying larval shell formation remain largely unexplored [8]. Presently, researchers have identified two different sources of cells as being involved in shell formation. The first source is the outer mantle epithelium that produces the organic matrix, including SMPs, which are responsible for controlling the shapes of the shells. The second source is hemocytes, which help deposit and transport calcium carbonate crystals to the mineralization site [9].

In shell biomineralization of mollusk, chitin and SMPs play a pivotal role [10]. Chitin (nitrogenous polysaccharide) is one of the major components of organic matter in most mollusks shells and plays a key role in the biomineralization of shell frameworks. The involvement of chitinase in the shell biomineralization process of oyster has been confirmed in a recent study [11]. The synthesis of SMPs take place in the mantle tissue and secreted into the space between the mantle and shell (extrapallial). In extrapallial space, SMPs interacts bicarbonate ions (HCO_3_^−^), calcium (Ca^2+^), trace metals, and polysaccharides. These interactions lead to the development of aragonite and calcite microstructures [12]. To date, the structure and function of several SMPs have been characterized [13,14], although the mechanisms of shell-building processes are still largely unidentified.

Additionally, a large number of mollusk SMPs, which are involved in the formation of the nacreous layer have been identified, such as Nacrein, Pif177, Pif, N19, N14, N66, N40, N16/pearlin, perlucin, perlustrin, lustrin A, P10, AP7, AP24, MSI60, MSI31, mucoperlin, perlwapin, perlinhibin, and blue mussel shell protein (BMSP). Conversely, prismalin-14, prisilkin-39, KRMPs, aspein, P43, Alv, and asprich proteins have been isolated from the calcitic prism texture and shown to be indispensable in the creation of prismatic layer.

During molluscan larval development, the trochophore larvae’s shell field secretes the first shell, which subsequently forms either round or D-shaped shells after 18 to 25 h of fertilization [15]. Thereafter, adult shells are formed following the settlement and metamorphosis of the veliger larvae into juveniles [16].

The abalone shell has served as a prominent model for studying the fundamental mechanism of gastropod shell biomineralization. Proteomic and transcriptomic analyses have revealed numerous novel gene sequences related to shell formation, with most of them being found in bivalves [17]. In contrast, very few proteins have been identified in *Haliotis rufescens*, *Haliotis laevigata*, and *Haliotis asinina* using proteomic approaches [15]. All these proteins were isolated from the adult abalone shell, and their role in the biomineralization process has been demonstrated in vitro by some studies [18,19]. The SMP extracted from the nacreous layer in the red abalone shell was Lustrin A [20].

However, following the advancement of technology and increased research activity, scientists are discovering the involvement of new genes in shell biomineralization. Recently, transcriptome analysis of some larval and adult bivalve and gastropod mollusk genomes identified SMPs, such as SMP1, SMP2, SMP3, SMP4, SMP5, SMP6, and SMP7. It is understood that substantial morphological, microstructural, and mineralogical alterations take place in the shell, beginning with the early stages of biomineralization in larvae, progressing through metamorphosis and juvenile growth, and ultimately resulting in the formation of the fully developed adult shell [21,22,23]. Despite the significant ontogenic changes observed in shell formation, the molecular mechanisms responsible for these processes are still largely unidentified and are just beginning to be clarified in mollusks [24].

Even though over a thousand SMPs with diverse structures have been identified from mollusk shells through proteomic methods, only a small number of them have been fully characterized. This is primarily due to the limited presence of matrix proteins on the shell surface, making it extremely challenging to obtain an adequate quantity of protein samples from these sources. Environmental stressors can induce physiological changes in cultured animals, impacting their growth, reproduction, metabolism, and osmotic pressure control. Thermal stress, in particular, can lead to various issues, including disease outbreaks and increased mortality in invertebrates due to its impact on their activity range. Previously, differential shell growth was observed in *Pinctada margaritifera* due to nutritional and temperature differences. Starvation was also previously reported to affect larval shell growth in clam species. Thus, the aim of the present study was to isolate SMP5 and analyze expression to highlight that SMP5 may play a role in shell biomineralization in infant and adult *H. discus hannai* and its regulation under antinutritional and thermal stresses.

## 2. Materials and Methods

### 2.1. Experimental Animals

Three-year-old Pacific abalones (male and female), with an average body weight of 120.4 ± 0.61 g and an average shell length of 84.06 ± 0.32 mm, were collected from sea cages in Jindo-gun, Korea. The collected abalones were then transported using oxygenated water car to the Tou-Jeong Soosan abalone hatchery in Yeosu, Korea, where they were cultured in tanks for 15 days with a continuous flow of seawater, and an adequate feed (kelp) supply for adjustment. The water quality in the tank was maintained with a temperature of 19.4 ± 2.3 °C, pH of 7.53 ± 0.22, dissolved oxygen of 8.38 ± 2.36 ppm, and salinity of 31 ± 1.19 PSU during this adjustment period.

### 2.2. Tissue Collection for Gene Cloning and Expression Analysis

For the cloning purpose, a total of 12 Pacific abalones were euthanized, and tissue samples were collected from various parts of their bodies, including hemocytes (HMC), cerebral ganglion (CG), testis (TES), ovary (OVR), mantle (MNT), muscle (MUS), gill (GIL), and digestive gland (DG). Before the tissue collection, the abalones were anesthetized using a 5% MgCl_2_ solution. Subsequently, the collected tissue samples were rinsed with phosphate-buffered saline (PBS; 0.1 M), promptly frozen by immersion in liquid nitrogen (LN_2_), and then stored at −80 °C until total RNA extraction.

### 2.3. Embryo and Larvae Collection

Three-year-old reproductively mature female and male abalone were induced for artificial fertilization. Among them, three females and two males were responded to spawn. After spawning, artificial fertilization was conducted following the procedure described previously [25]. After fertilization, samples of fertilized eggs (FE), 2-cell (2-CL) and 4-cell (4-CL) stages, morula (MOR), trochophore (TRP) larvae, veliger (VLG) larvae, and juveniles (JUV) were collected through microscopic observation. The samples were immediately flash-frozen in LN_2_ and stored at −80 °C until total RNA extraction.

### 2.4. Tissue Collection during Shell Biomineralization

Ten-month-old juvenile Pacific abalones were used in the shell biomineralization experiment. Juvenile abalone (*n* = 40) were divided into two groups: group-1 served as the control, and group-2 underwent shell damage treatment. The shell of each abalone in group-2 was punctured (1 mm round orifice) near the mantle tissue using a perforator, and then cultured in a rearing tank with continuous aeration, water, and food supplied. During the experiment, the water temperature of the tank was 19.4 ± 2.1 °C. Mantle tissues were collected from 5 individuals weekly. Mantle tissue from the control abalone was also collected at the beginning of the experiment. After collection, the tissues were rinsed with PBS, rapidly frozen in LN_2_, and kept at −80 °C until extraction of the total RNA.

### 2.5. Tissue Samples from Starved Pacific Abalones

Two-year-old Pacific abalones were randomly collected from sea cages located in Wando-gun, South Korea, and subsequently transported to an abalone hatchery in Yeosu, Korea. These collected abalones were then cultured in tanks for a period of 30 days with a continuous flow of seawater and sufficient food supply for their acclimatization. Then, abalones (*n* = 48) were divided into two groups: group-A served as the control, and group-B underwent starvation treatment. Thereafter, mantle tissues were collected from three Pacific abalones from group-A after anesthetizing them using the procedure outlined earlier and then stored for the extraction of total RNA. The group-B abalones (*n* = 24) were cultured without providing food, and mantle tissue was collected at intervals of one week for three weeks on the next day after re-feeding had occurred and stored at −80 °C until total RNA extraction, using the procedure mentioned above. Samples from control treatment were also collected each time point.

### 2.6. Tissue Samples from Heat-Stressed Pacific Abalones

To characterize the changes in *Hdh-SMP5* expression in Pacific abalones in response to stress, heat treatments were performed at 15 °C, 25 °C, and 30 °C. For this experiment, three-year-old Pacific abalones, with a mean weight of 121.06 ± 0.41 g and a shell length of 82.8 ± 0.52 mm, were collected from the sea cage abalone culture area in Wando-gun and transported to the Tou-Jeong Soosan abalone hatchery in Dolsan-eup, Yeosu, South Korea. To recover from any transportation-related stress and to adjust to the hatchery environment, the abalones were left to acclimatize in tanks for about 20 days with a sufficient food supply. Thereafter, 12 abalones were kept in three different aquariums for 24 hours each. For the 25 and 30 °C treatment tanks, the water temperature was gradually increased (2–4 °C/h) to avoid sudden heat shock using a digital temperature controller. During this experiment, the abalones were not provided with any food. Thereafter, the mantle tissue from five individuals was collected from each treatment and controlled abalone at 1, 6, 12, and 24 h, washed with PBS (0.1 M), flash-frozen in LN_2_, and stored at −80 °C until total RNA extraction. The temperature in the control tank was 20.4 °C.

### 2.7. Total RNA Extraction and First-Strand cDNA Synthesis

The total cellular RNA from the collected tissue samples was extracted using the ISOSPIN Cell and Tissue RNA Kit (Nippon Gene, Tokyo, Japan). First-strand cDNA synthesis was performed using extracted RNA, oligo(dt) primers (Sigma, Burlington, MA, USA), and the Superscript III First-Strand cDNA Synthesis Kit (Invitrogen, Carlsbad, CA, USA). The RACE cDNA (3′- and 5′-RACE) was synthesized from the extracted total RNA using the SMARTer^®^ RACE 5′/3′ Kit (Takara Bio Inc., Kusatsu-Shi, Japan). Both the total RNA extraction and cDNA synthesis procedures were carried out following the guidelines provided by the manufacturer.

### 2.8. Cloning of SMP5 mRNA Sequence in Pacific Abalone

#### 2.8.1. Cloning of Partial Sequence

Reverse transcription polymerase chain reaction (RT-PCR was conducted for partial sequence cloning of Hdh-SMP5 using muscle tissue cDNA, gene-specific forward and reverse primer, and GoTaq^®^ DNA polymerase (Promega, Madison, WI, USA). The gene-specific primer used in the present study was designed from the known *H. rufescens* SMP5 mRNA sequence (GenBank Accession no. XP_046328359.1). All primers used in this study are enlisted in Table 1. The RT-PCR reaction mixture was assembled in a final volume of 50 μL. It included 1 μL of cDNA, 1 μL each of the forward (Hdh-SMP5 Fw) and reverse (Hdh-SMP5 Rv) primers (forward and reverse) 1 μL each, 10 μL of colorless GoTaq reaction buffer, 1 μL of dNTP mix, 0.25 μL of DNA polymerase, and 35.75 μL of ultra-pure water. The thermal cycling parameters for the RT-PCR process comprised an initial denaturation step lasting 3 min at 95 °C. This was followed by 35 cycles, each consisting of denaturation at 95 °C for 30 s, annealing at 58 °C for 30 s, and extension at 72 °C for 45 s. The reaction concluded with a final extension step at 72 °C for 5 min. After completion, the PCR products were subjected to gel electrophoresis using 1.2% agarose, after which bands were purified using the Wizard^®^ SV Gel and PCR Clean-Up System kit (Promega, Madison, WI, USA). Afterward, the purified DNA was ligated into the pGEM^®^-T Easy Vector (Promega, Madison, WI, USA) and transformed into DH5α chemically competent *E. coli* cells (Enzynomics, Daejeon, Korea). Positive clones were chosen for plasmid DNA purification, which was carried out utilizing a Hybrid-QTM Plasmid Rapidprep mini kit (GeneAll, Seoul, Korea). Subsequently, the purified plasmid DNA samples were subjected to sequencing at Macrogen (Seoul, Korea).

#### 2.8.2. Cloning of RACE (5′- and 3′) Sequence

To obtain the complete sequence of Hdh-SMP5, 5′- and 3′-RACE-PCR (rapid amplification of cDNA ends polymerase chain reaction) was conducted. The SMARTer^®^ RACE 5′/3′ kit from Takara Bio Inc. (Japan) was employed for this purpose. A pair of gene-specific 5′- and 3′-RACE primers were custom-designed based on the partial sequence of Hdh-SMP5 that had been previously obtained. Reaction mixtures for RACE PCR (3′- and 5′-RACE) were prepared using 2.5 μL cDNA (3′- or 5′-RACE), 1 μL RACE primers, 1 μL SeqAmp DNA polymerase, 5 μL universal primer mix (UPM), 25 μL SeqAmp buffer, and 15.5 μL ultra-pure water. Thirty cycles of touchdown PCR were carried out for RACE-PCR. The thermal cycle conditions were set following the kit manufacturer’s instructions. Upon completion of the PCR amplification, the resulting products were subjected to gel electrophoresis, following the previously described procedure. Subsequently, the positively identified bands were purified using the NucleoSpin^®^ Gel and PCR Clean-up kit, manufactured by MACHEREY-NAGEL GmbH & Co. KG (Düren, Germany). After purification, the obtained products were ligated into a linearized pRACE vector and subsequently introduced into Stellar competent cells (*E. coli* HST08) through transformation. Positive clones were isolated and subjected to sequencing at Macrogen, using the same procedures as described for the partial sequence. Finally, the 5′-RACE sequence, the initially cloned partial cDNA fragment, and the 3′-RACE sequence were integrated and refined to obtain the full-length sequence.

### 2.9. Sequence Analysis of Cloned H. discus Hannai SMP5 (Hdh-SMP5)

The full nucleotide and amino acid sequences of the cloned Hdh-SMP5 were subjected to analysis using different online tools. The ORFfinder tool, available at https://www.ncbi.nlm.nih.gov/orffinder/ (accessed on 12 June 2022), was used to predict potential protein-encoding segments and open reading frames (ORFs) from the nucleotide sequence. The ProtParam tool (https://web.expasy.org/protparam/) (accessed on 28 March 2023) was utilized to predict the molecular weight, isoelectric point (pI), amino acid composition, and stability index of the Hdh-SMP5 protein sequence. SignalP 6.0 (https://services.healthtech.dtu.dk/services/SignalP-6.0/) (accessed on 4 April 2022) was employed to identify signal peptides in the amino acid sequence. The protein solubility was predicted using the server https://protein-sol.manchester.ac.uk/ (accessed on 4 April 2023). The online protein function and structure prediction server C-I-TASSER (https://zhanggroup.org/C-I-TASSER/) (accessed on accessed on 6 April 2023) was used to analyze and predict the three-dimensional (3D) structure and function of the Hdh-SMP5 protein. The motif scan tool (https://myhits.sib.swiss/cgi-bin/motif_scan) (accessed on 6 April 2023) was utilized to predict any motifs present in the Hdh-SMP5 sequence. Multiple sequence alignments were performed using MEGA (version 11.0.13), to compare the amino acid sequence of Pacific abalone SMP5 with other abalone species, after which the results were visualized using Jalview (version 2.11.1.7).

### 2.10. Phylogenetic Analysis

The amino acid sequence of Hdh-SMP5 was aligned with sequences of other SMP5 proteins and related SMPs, yet uncharacterized protein sequences using the Clustal Omega online tool (https://www.ebi.ac.uk/Tools/msa/clustalo/, accessed on 8 April 2022). Subsequently, a phylogenetic tree was constructed using the Maximum Likelihood algorithm within the MEGA software (version 11.0.13). Finally, phylogenetic tree was visualized and edited using iTOL online tool (https://itol.embl.de/) (accessed on 8 April 2022).

### 2.11. Quantitative Real-Time PCR (qRT-PCR) Analysis

The relative mRNA expression level was calculated by performing qRT-PCR analysis on various Pacific abalone tissues. The *Hdh-SMP5* expression levels were measured in adult abalone organs, early developmental stages, shell biomineralizing sample, as well as stress treatment (heat stress and starvation) samples. All qRT-PCR assays were conducted according to the 2 × qPCRBIO SyGreen Mix Lo-Rox kit (PCR Biosystems Ltd., London, UK) manual. Each qRT-PCR reaction mixture was prepared in a total volume of 20 μL containing cDNA template (1 μL), 10 pmol gene-specific forward and reverse primer (1 μL each), SyGreen Mix (10 μL), and double distilled water (10 μL). Triplicate reactions were performed for target and reference genes in each tissue sample. The PCR amplification conditions consisted of a preincubation step at 95 °C for 2 min, followed by 40 cycles of denaturation, annealing, and extension at 95 °C for 30 s, 60 °C for 20 s, and 72 °C for 30 s. The melting temperature was set as the instrument’s default setting. At the end of each cycle, a fluorescence reading was recorded for quantification. A LightCycler^®^ 96 System (Roche, Mannheim, Germany) was used for amplification and data analysis. The relative gene expression was determined using the 2^−ΔΔCT^ method with the Pacific abalone β-actin gene (accession no. MW387000) as an internal reference. All primers used in qRT-PCR analysis are summarized in Table 1.

### 2.12. Statistical Analysis

The mRNA expression values were subjected to statistical analysis and presented as the mean ± standard error. Changes in relative mRNA expression were calculated by nonparametric ANOVA analysis using GraphPad Prism software (version 9.3.1). The statistical significance level was set at *p* < 0.05, *p* < 0.01, and *p* < 0.001. All graphs were generated using Microsoft Excel and the GraphPad Prism 9.3.1 software.

## 3. Results

### 3.1. Hdh-SMP5 Sequence Analysis

**Figure 1:**
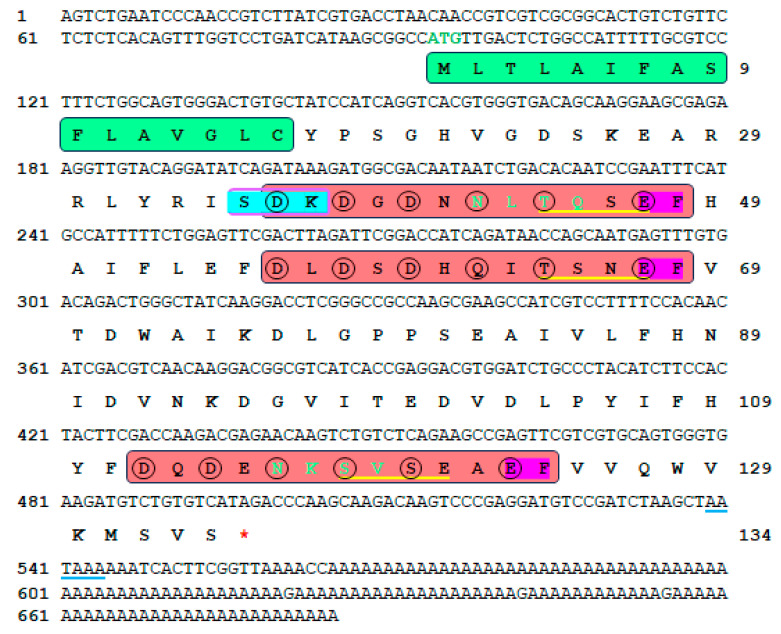
Full-length Hdh-SMP5 nucleotide and deduced amino acid sequences (GenBank accession No. ON803450.1). The nucleotides and amino acids are indicated by the numbers on the left and right sides of the sequence, respectively. The start and stop codons are indicated in green bold font and by a red asterisk, respectively. The signal peptide is indicated by the green box. The PKC phosphorylation site is denoted by a cyan box. Predicted casein kinase II phosphorylation sites are indicated in yellow underlines. The spring-green letter represents the N-glycosylation sites. The N-myristoylation sites are indicated in orange. The red boxes indicate the Ca^2+^ binding domain with EF-hand in violet color. The black circles indicate the calcium-binding residues. The underlined bases are the putative polyadenylation signal.

The cDNA sequence encoding H. discus hannai SMP5 was cloned from the mantle tissue of H. discus hannai and designated as Hdh-SMP5. The full-length Hdh-SMP5 cDNA sequence (GenBank accession No. ON803450.1) was 685 bp long, including the poly-A tail (Figure 1), and its 5′- and 3′-untranslated regions (UTRs), which were 93 bp and 187 bp long, respectively. A putative polyadenylation signal (AATAAA) was identified in its nucleotide sequence approximately 20 bp upstream of the poly-A tail. The Hdh-SMP5 cDNA sequence ORF was 405 bp, with 134 deduced amino acids (Figure 1). Amino acid composition analysis showed that Hdh-SMP5 is rich in aspartic acid, serine, and valine (supplementary Table S1). Finally, Hdh-SMP5 was confirmed as a soluble SMP by protein solubility analysis.

The first sixteen residues in the amino acid sequence are detected as signal peptide. A total of five phosphorylation sites were observed, which included one protein kinase C (PKC) phosphorylation site [S/T]-X-[R/K] at positions 35S–K37, and four casein kinase II phosphorylation sites [S/T]-X(2)-[D/E] at 35S–D38, 44T–47E, 64T–67E, and 118S–121E. Additionally, two potential N-glycosylation sites were noted at positions 42N–45Q and 116N–119V (Figure 1). Furthermore, sequence analysis was used to predict three Ca^2+^-binding domains with EF-hand domains within the amino acid sequence at positions 36-48, 56–68, and 112–124.

**Figure 2:**
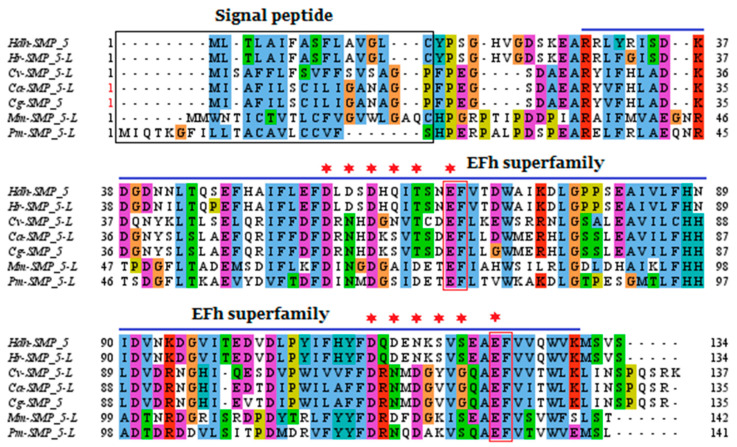
Multiple sequence alignment of Hdh-SMP5 amino acid sequence. Signal peptide indicated by the black box. The blue line depicts the conserved domain (EFh superfamily) of Hdh-SMP5. The asterisk marks are for the conserved Ca^2+^-binding sites and the red box presents the conserved EF-hands that bind Ca^2+^. The following protein sequences with their accession id were used to construct the phylogenetic tree: H. discus hannai (UYL69037), Haliotis rufescens (XP_046328359), Crassostrea virginica (XP_022323531), Crassostrea angulata (XP_052720920), Crassostrea gigas (XP_034301324), Mercenaria mercenaria (XP_045199821), and Pecten maximus (XP_033732420).

In the multiple sequence alignment, the first 16–21 residues in the N-terminal region were detected as signal peptides, when aligned with SMP5 of other molluscan species (Figure 2), (*C. angulata* and *C. giggas* do not possess signal peptide). In the EFh superfamily domain, two EF-hand–Ca^2+^ regions were conserved among the species analyzed for multiple sequence alignment; however, the binding residues were found to be different. Overall, the SMP5 C-terminal region was found to be more comparatively conserved than the N-terminal region of the analyzed protein sequences.

### 3.2. Structure of Hdh-SMP5

**Figure 3:**
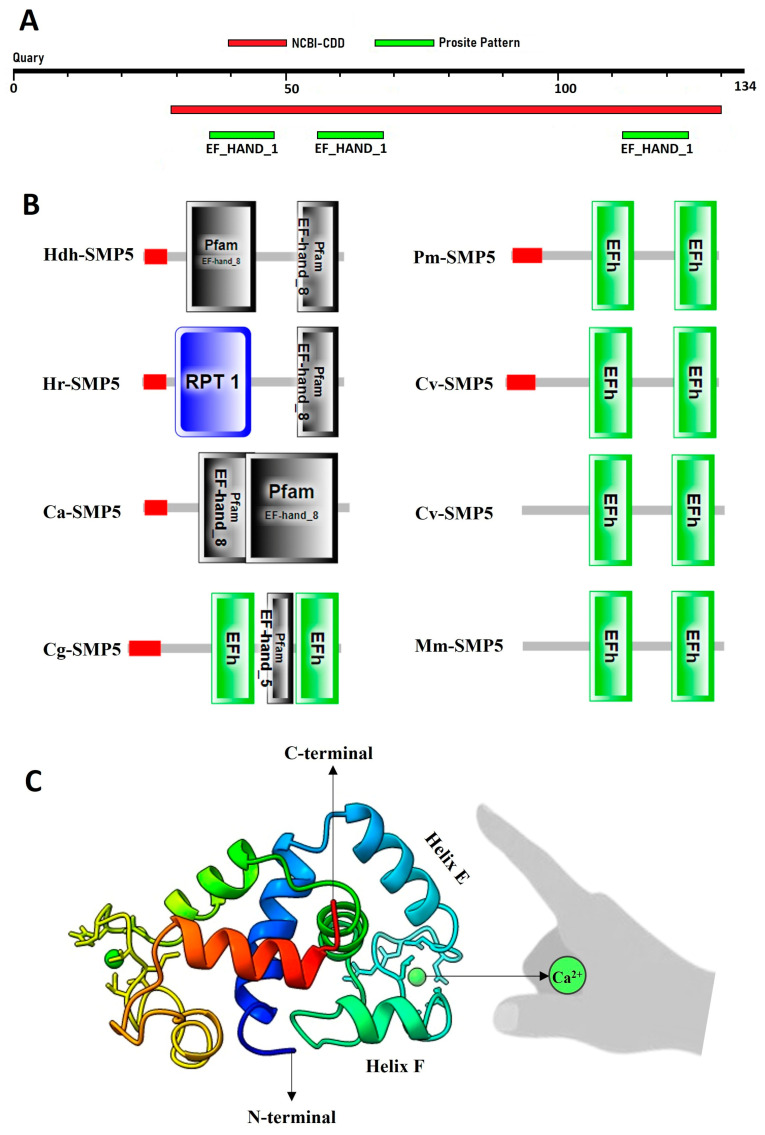
Domain architecture of SMP 5 in Pacific abalones and other mollusk species: (**A**) NCBI-conserved domain and PROSITE pattern (conserved Ca^2+^-binding domain); (**B**) Pfam domain with other SMP5 protein sequences; (**C**) A 3D structure with EF-hand Ca^2+^ binding feature.

Structurally, Hdh-SMP5 is an EF-hand-binding protein. Further, the NCBI-CDD confirmed Hdh-SMP5 as a member of the EF-hand-binding superfamily. The PROSITE pattern detected three EF-hand Ca^2+^-binding domains in the protein sequence (Figure 3A). Alternatively, the Pfam domain analysis mainly detected the signal peptide and EF-hand-binding domain-8 in Hdh-SMP5 (Figure 3B). However, Pfam also detected other domains, although it depended on the species, such as the internal repeat (Hr-SMP5), the EF-hand-binding domain-5 (Mm-SMP5), and the EFh domain.

The secondary structure of Hdh-SMP5 was dominated by coiled–coil helices and an acidic region, yet with no beta-strands (Figure 3C). Although the amino acid sequence analysis found three EF-hand calcium-binding domains, the three-dimensional structure predicted only two Ca^2+^ ligands (one in the N-terminal region and another in the C-terminal region).

### 3.3. Phylogenetic Analysis

**Figure 4:**
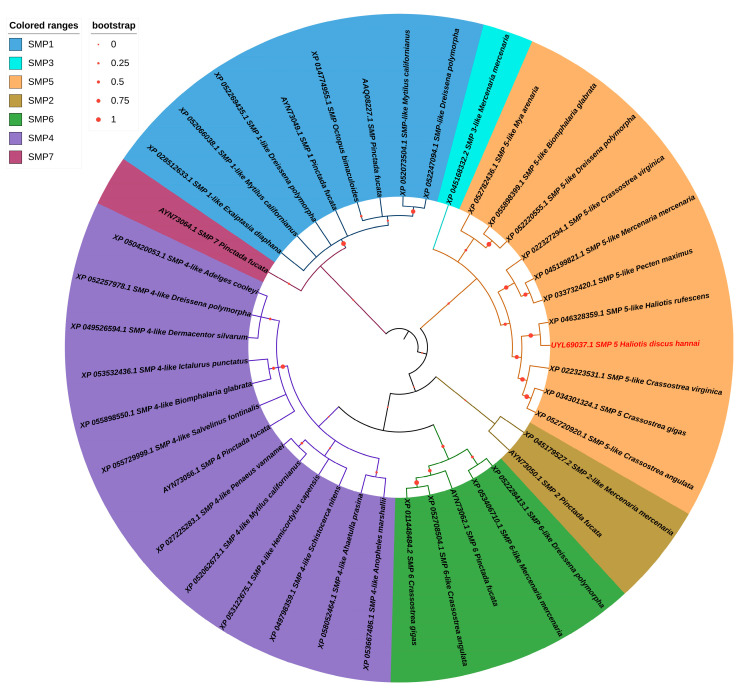
The phylogenetic tree of SMPs was generated through the maximum likelihood method after performing a ClustalW alignment using amino acid sequences from various vertebrate and invertebrate species. Different clade colors in the tree indicate representative shell matrix proteins on the left side of the tree.

The constructed phylogenetic tree, based on the amino acid sequence and using the maximum likelihood method, formed seven different clusters for SMP1, SMP2, SMP1, SMP3, SMP4, SMP5, SMP6, and SMP7. Among the seven SMPs, all were reported from invertebrates and dominated by bivalve and gastropod mollusks, except SMP4, which was also reported from a vertebrate (Actinopterygii) species. Moreover, the vertebrate species in the SMP4 cluster formed a separate subcluster. In the SMP5 cluster, Hdh-SMP5 was closely related to Hr-SMP5 and constructed a subtree (Figure 4). SMP2, SMP3, and SMP7 have only been reported in one species.

### 3.4. Functional Activity Prediction (Gene Ontology)

A gene ontology (GO) analysis found that Hdh-SMP5 is involved in calcium ion (Ca^2+^) binding and catalytic activity and in terms (Figure S2A) of its molecular function (GO: 0003674). In addition, it was associated with signal transduction, cellular protein metabolic process, and also response to stimulus (environmental stimuli) in biological processes (GO: 0008150) (Figure S2B). In terms of cellular components (Figure S2C), Hdh-SMP5 was associated with intracellular part and nucleus (GO: 0005575).

### 3.5. Tissue-Specific mRNA Expression of Hdh-SMP5

**Figure 5:**
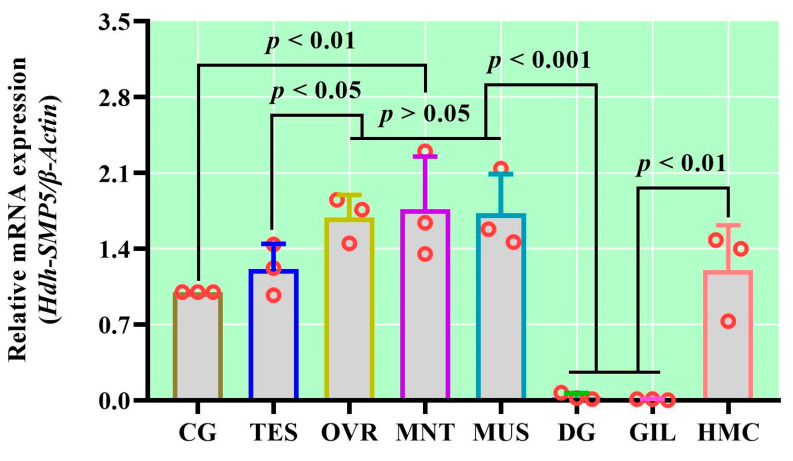
The mRNA expression levels of *Hdh-SMP5* in various tissues of Pacific abalone. Cerebral ganglion (CG), testis (TES), ovary (OVR), mantle (MNT), muscle (MUS), digestive gland (DG), gill (GIL), and hemocyte (HMC). Different *p*-value in the figure indicates the level of significance.

Among the analyzed tissues, Hdh-SMP5 mRNA was most significantly (*p* < 0.05) expressed in the mantle of the Pacific abalone, followed by the muscles, ovaries, hemocyte, testis, and ganglion (cerebral) (Figure 5). The lowest expression of Hdh-SMP5 mRNA was observed in the digestive gland and gill tissues. These findings were consistent with those of our semi-quantitative RT-PCR expression analysis (Figure S1).

### 3.6. Hdh-SMP5 Expression in Pacific Abalone during Shell Biomineralization

**Figure 6:**
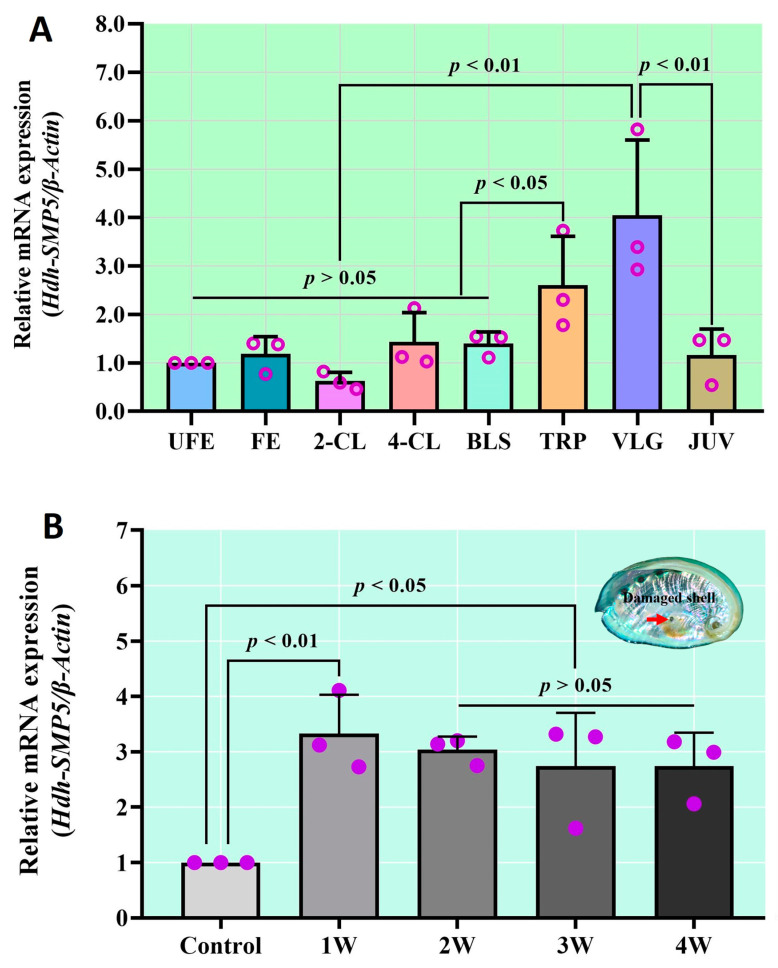
(**A**) Hdh-SMP5 mRNA expression in Pacific abalone during early shell formation; (**B**) Hdh-SMP5 expression during injured shell regeneration. Unfertilized eggs (UFEs), fertilized eggs (FEs), 2-cell (2-CL) and 4-cell (4-CL) stages, Blastula (BLS), trochophore (TRP) larvae, veliger (VLG) larvae, and juveniles (JUV). First week (1W), second week (2W), third week (3W), fourth week (4W). Different *p*-value in the figure indicates the level of significance.

A dynamic expression of Hdh-SMP5 mRNA was observed during the embryonic and larval development stages. After fertilization, no significant differences were observed until the blastula stage in the control (unfertilized egg). However, expression increased in the trochophore stage, and the most significant (*p* < 0.05) expression was found in the veliger stage (Figure 6A). In the juvenile stage, the Hdh-SMP5 expression was similar to the controls.

Figure 6B illustrates the expression of Hdh-SMP5 mRNA during the Pacific abalone shell biomineralization. In the first week following damage to the shell, the expression was significantly higher than in the control abalones. Afterward, a gradual decrease in the expression was observed over the remaining experimental period, although not to levels of significance. However, the Hdh-SMP5 expression was significantly higher than in the control abalone during the study period.

### 3.7. Hdh-SMP5 Expression during Starvation

**Figure 7:**
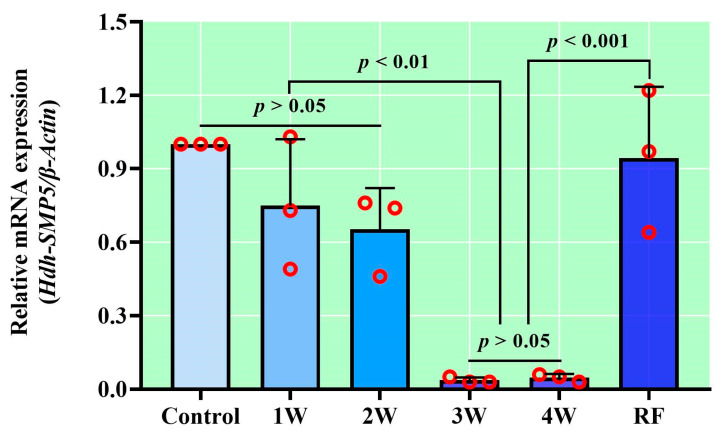
Hdh-SMP5 expression in mantles of starved Pacific abalone H. discus hannai. First week (1W), second week (2W), third week (3W), fourth week (4W), re-feeding (RF). Different *p*-value in the figure indicates the level of significance.

Under starvation, which induce nutritional stress, notable variations in Hdh-SMP5 mRNA expression were detected within the mantle tissues. The expression level decreased with starvation until re-feeding occurred, with significant reductions observed in the expression during the third and fourth weeks of starvation (Figure 7). However, the Hdh-SMP5 expression in the mantle returned to levels similar to the control after re-feeding.

### 3.8. Hdh-SMP5 mRNA Expression in Heat-Stressed Pacific Abalones

**Figure 8:**
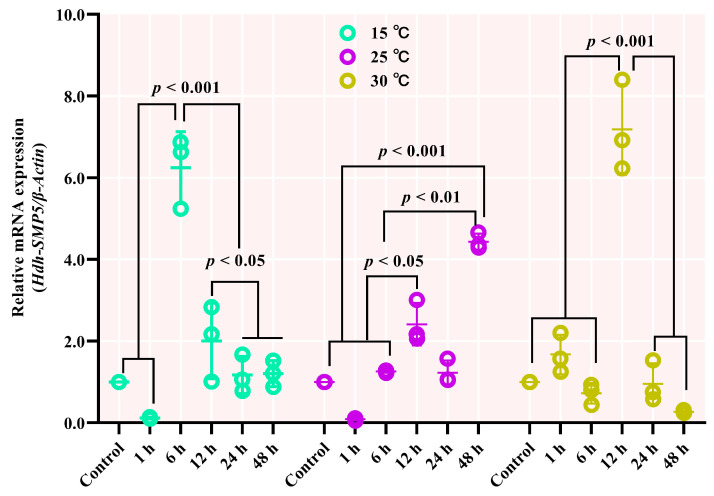
Hdh-SMP5Pinc expression during thermal stress of Pacific abalone *H. discus hannai*. Different *p*-value in the figure indicates the level of significance.

Under thermal stress, the Hdh-SMP5 mRNA was differentially expressed depending on temperature. The expression of Hdh-SMP5 at 15 °C decreased after 1 h but significantly increased after 6 h. However, the expression again declined significantly after 12 h before remaining constant, after a negligible reduction after 24 h of thermal stress. At 25 °C, an insignificant up and down change in the expression of Hdh-SMP5 was observed until 24 h, although at the 48 h time point, the expression was significantly higher. However, after 1 h of heat treatment at 30 °C, the mRNA expression of Hdh-SMP5 was increased compared to the control, although not to levels of significance. Thereafter, it again decreased after 6 h before significantly increasing at 12 h, and again decreasing at 24 h. Finally, after 48 h of treatment, it reached levels lower than those observed in the control treatment (Figure 8).

## 4. Discussion

The mollusk shell comprises a complex structure composed of both organic and mineral constituents. SMPs have been identified as the primary organic components with pivotal roles in the development of mollusk shells, which consist of layers composed of calcium carbonate polymorphs, including aragonite or calcite crystals [26,27]. Different soluble and insoluble SMPs form calcium carbonate polymorphs [28,29]. Insoluble proteins are chitin–protein complexes characterized by an abundance of aliphatic amino acids like isoleucine (Ile), leucine (Leu), valine (Val), and alanine (Ala). In contrast, soluble proteins exhibit polyanionic properties and are enriched in aspartic acid (Asp), playing a role in crystal nucleation [5]. The Hdh-SMP5 protein in the Pacific abalone mantle tissue is rich in Asp (11.2%) and contains a signal peptide, thereby indicating a secreted soluble acidic protein. The amino acid composition analysis performed in previous studies has shown that glycine, alanine, serine, and aspartic acid were richer in the nacreous layer than other amino acids [30]. The Hdh-SMP5 in the Pacific abalone mantle tissue is rich in Asp (11.2%) and serine (9%); thus, it may function in the formation of the nacreous layer.

The pioneering work on the organic matrix of shells confirmed the existence of acidic proteins in molluscan shells [31]. The Hdh-SMP5 has a pI of 4.31 and is acidic in nature. Acidic proteins are also found in *C. giggas* larval shells, in addition to the larval shell of *Pinctada fucata*. Indeed, three novel proteins with extremely acidic natures (pI < 4.5) were found that are rich in aspartic acid and pIs ranging from 3.8 to 4.4 [2]. Shell-forming acidic proteins have side chains with a negative charge that are rich in aspartic and/or glutamic acid and possess calcium-binding ions [26,32]. The Hdh-SMP5 has 24 negatively charged amino acid (aspartic acid and glutamic acid) residues and 9 positively charged amino acid (arginine and lysine) residues with calcium ion binding activity. Until now, only a few acidic SMPs have been identified [33], due to technical difficulties in isolating these acidic proteins [34].

The conserved functional domains across SMPs signifies their essential function in the process of shell formation. The SMPs with EF-hand domains have been extensively studied and observed in various living organisms. These proteins play a crucial role in regulating a wide range of functions, such as calcium buffering, transport, signal transduction, and muscle contractions [35]. For example, the presence of conserved functional domains, such as the EF-hand calcium-binding domain, plays a pivotal role in the regulation of shell biomineralization [36]. Hdh-SMP5 belongs to the EFh superfamily and contains three calcium-binding EF-hand domains. Recently, three novel SMPs were observed in the larval shell of pearl oysters containing calcium-binding EF-hand domains [2]. In Pacific oysters (*C. giggas*), SMPs with the EF-hand calcium-binding-5 domain were also reported in larval shells. EF-hand proteins from the bivalve mantle tissue act as signal modulators and induce conformational changes by binding Ca^2+^. The Hdh-SMP5 protein may be involved in signal transduction during this process, leading to the biomineralization of nacre when it binds Ca^2+^.

The mantle is the most important biomineralization tissue in mollusks. Since shell matrix proteins are secreted by mantle epithelial cells, highly expressed genes in the shell reflect their involvement with shell formation. During expression analysis, *Hdh-SMP5* mRNA was significantly expressed in the Pacific abalone’s mantle tissue. Various regions within the mantle are responsible for the secretion and expression of matrix proteins and regulatory factors, all of which play integral roles in the crystallization of the three distinct shell layers: the periostacum, prismatic layer, and nacreous layer. Earlier research has unveiled that proteins expressed within the epithelial cells of the mantle pallial, including nacrein, MSI60, Pif, lustrin A, N16/pearlin, perlucin, perlustrin, N14, N66, mucoperlin, AP7, AP24, P10, perlwapin, perlinhibin, N19, and N40, collectively contribute to the formation of the nacreous layer; the proteins from the outer and inner epithelial cells of the outer fold, such as prisilkin-39, Prismalin-14, MSI31, P43, Aspein, Alv, KRMP, and asprich proteins, design the prismatic layer structure [7]; the proteins from the outer epithelial cells of the middle fold, such as OT47, HcTyp-1, and tyrosinase [37], are responsible for the periostracum formation.

In gastropods, the shell formation process begins during embryonic development, starting with the creation of the shell gland responsible for secreting the periostracum. Subsequently, the shell gland undergoes eversion to generate the shell field, which eventually forms the calcifying mantle. The adjacent epithelial cells produce a delicate organic layer, from which the initial larval organic shell originates. Generally, in abalones, the shell glands form at the end of the morula stage, and the epithelial cells bordering the shell are detected in the trochophore stage. During the early veliger stage, the larval mantle initiates calcium carbonate (CaCO_3_) precipitation by synthesizing and releasing organic components [5]. During the expression analysis of *Hdh-SMP5*, a significantly higher expression was found in the trochophore and D-veliger larvae stages, which indicates its potential involvement in the early formation of the shell. Recent studies on the mineralogical composition of the *H. tuberculata* shell from the early protoconch to the juvenile found that the early protoconch was mostly composed of amorphous calcium carbonate, while the shells of veliger larvae to juveniles were essentially made of aragonite [22,23]. Remarkably, in situ hybridization of the fully developed adult shell revealed a combination of prismatic calcite and aragonitic spherulitic prismatic structures in the outermost layer. Furthermore, it has been demonstrated that mineralization takes place during this larval stage in abalones, and the mineral phase initially deposited is predominantly composed of aragonite [22]. Out of the 21 gene models, 12 genes were identified as larval SMP genes, and their expression was observed during the trochophore and/or D-shaped stages, thus providing confirmation of their involvement in the formation of the larval shell. In adults, acidic SMPs are rich in aspartic acid [5], contain calcium-binding domains, and are known to nucleate CaCO_3_ crystals [14].

A few studies have revealed the relationship between shell matrix proteins and shell microstructures during shell biomineralization. Previously, the soluble shell matrix protein *HyTyp-1* was found to be involved in periostracum formation [38]. In a recent study, during shell regeneration in freshwater mussels, the expression of the *HcTyp-1* SMP was found to be significantly increased compared to the control and was predicted to be involved in shell regeneration (nacreous layer biomineralization) [39]. During shell regenerations, the expression of *Hdh-SMP5* was significantly increased, which is in line with the abovementioned study and indicates the involvement of Hdh-SMP5 in the regeneration of shells by Pacific abalones. Previously, the expression of a shell matrix protein SPARC mRNA was reported to increase in the extrapallial fluid of *Pinctada fucata* after shell-notching [40], thereby suggesting its participation in the shell repair process.

Since the expression of *Hdh-SMP5* is reduced during starvation, it may have a negative impact on the shell growth of Pacific abalone. During starvation, the supply of Ca^2+^ from food is disrupted, which can lead to a reduction in protein activity and gene expression. For CaCO_3_ crystallization, Ca^2+^ is required, which comes from food and minerals. However, an increase in the length of the carpet-shell clam larvae shell was observed during starvation [41]. Body reserves and the catabolism of tissues provide energy during starvation. As a result, one of the initial outcomes of starvation is the depletion of organic matter, as witnessed in our present study during extended periods of starvation. A comparable decline in organic matter was also noted when either the quantity or quality of the available food was insufficient [42]. Since the expression of *Hdh-SMP5* was increased to control after refeeding, food may influence Hdh-SMP5 gene by supplying Ca^2+^ and other chemical components for its functionality. Numerous studies have demonstrated that both food and temperature are pivotal factors influencing the growth of somatic tissues and shells, primarily through their impact on SMPs [43]. Food regulates the level of transcription of the shematrin 9 and Pif 177 SMPs. The additional energy from food enhances the rate of shell deposition by involving these SMPs.

Hdh-SMP5 exhibited differential expression levels at various temperatures, attributable to thermal stress. The probable reason could be related to the unstable nature of Hdh-SMP5 protein because unstable proteins are generally affected by an alteration in the environmental temperature [44]. Extended exposure (48 h) to 30 °C resulted in a significant reduction (close to zero) in the expression of *Hdh-SMP5*, indicating that high temperatures negatively affect shell growth activity in Pacific abalone. Similarly, an extended exposure to a temperature of 28 °C decreased the expression levels of 8 of the 11 genes that encode for proteins involved in the shell matrix in *P. margaritifera*. In contrast, the MSI60, linkine, and shematrin 9 genes were not regulated by temperature. No effect was observed for temperatures between 27 and 30 °C on the expression of five mantle genes (calmodulin, aspein, nacrein, shematrin 7, and hsp70) in the pearl oyster *P. fucata* [45]; however, high temperature (30 °C) negatively affected the shell growth of *P. margaritifera* [46].

## 5. Conclusions

In summary, *Hdh-SMP5* has been cloned for the first time from the mantle tissue of Pacific abalone. The cloned Hdh-SMP5 possesses a signal peptide, indicating that it is a secreted protein. The presence of the EF-hand-binding structural domain, along with functional activities such as Ca^2+^-binding and catalytic activity, and its expression in mantle tissues suggest that this matrix protein, *Hdh-SMP5*, may play a role in the shell biomineralization of Pacific abalone. The higher expression of Hdh-SMP5 during the shell formation stages (trochophore and veliger) and throughout the shell damage experiment further supports this characteristic. Additionally, the differential expression of *Hdh-SMP5* during starvation and under thermal stress implies that this protein can be influenced by nutritional and thermal stress, subsequently impacting shell formation.

## Figures and Tables

**Table 1:** List of the various primers employed in this study for cDNA synthesis, cloning, and expression analysis.

Primer Name	Nucleotide Sequences	Purpose
Oligo dT (OdT)	GGCCACGCGTCGACTAGTACTTTTTTTTTTTTTTTTT	cDNA synthesis
Oligo dT adapter (AP)	GGCCACGCGTCGACTAGTAC	
SMP5-Fw	GATCATAAGCGGCCATGTTG	Fragment PCR
SMP5-Rv	CTTGCTTGGGTCTATGACAC	
SMP5-5′	GATTACGCCAAGCTTCACGTGACCTGATGGATAGCACAGTCC	5′ RACE PCR
SMP5-3′	GATTACGCCAAGCTTGGACTGTGCTATCCATCAGGTCACGTG	3′ RACE PCR
SMP5-qFw	TGACAGACTGGGCTATCAAG	qRT-PCR
SMP5-qRv	CTTGCTTGGGTCTATGACAC	
Hdh-β-Actin-Fw	GATAGTGCGAGACATCAAGG	
Hdh-β-Actin-Rv	GAGCTCGAAACCTCTCATTG	

## Data Availability

The data presented in this study are available in the manuscript and supplementary materials. The related raw data are available on request from the corresponding author.

## Supplementary Materials

The following supporting information can be downloaded at https://www.mdpi.com/article/10.3390/cimb45120629/s1.
