# Supplementary material for: EF-Hand-Binding Secreted Protein Hdh-SMP5 Regulates Shell Biomineralization and Responses to Stress in Pacific Abalone, Haliotis discus hannai
Source: Curr Issues Mol Biol. 2023 Dec 13;45(12):10079–96. doi: 10.3390/cimb45120629 (PMC10741955; doi:10.3390/cimb45120629)
Supplement: Supplementary file 1 [file cimb-45-00629-s001.zip › cimb-2743271-supplementary.pdf]

Table S1: Amino acid composition of Hdh-SMP5 in Pacific abalone

Amino acids	number	Percentage
Ala (A)	8	6.0%
Arg (R)	3	2.2%
Asn (N)	6	4.5%
Asp (D)	15	11.2%
Cys (C)	1	0.7%
Gln (Q)	4	3.0%
Glu (E)	9	6.7%
Gly (G)	6	4.5%
His (H)	5	3.7%
Ile (I)	9	6.7%
Leu (L)	11	8.2%
Lys (K)	6	4.5%
Met (M)	2	1.5%
Phe (F)	10	7.5%
Pro (P)	4	3.0%
Ser (S)	12	9.0%
Thr (T)	5	3.7%
Trp (W)	2	1.5%
Tyr (Y)	4	3.0%
Val (V)	12	9.0%

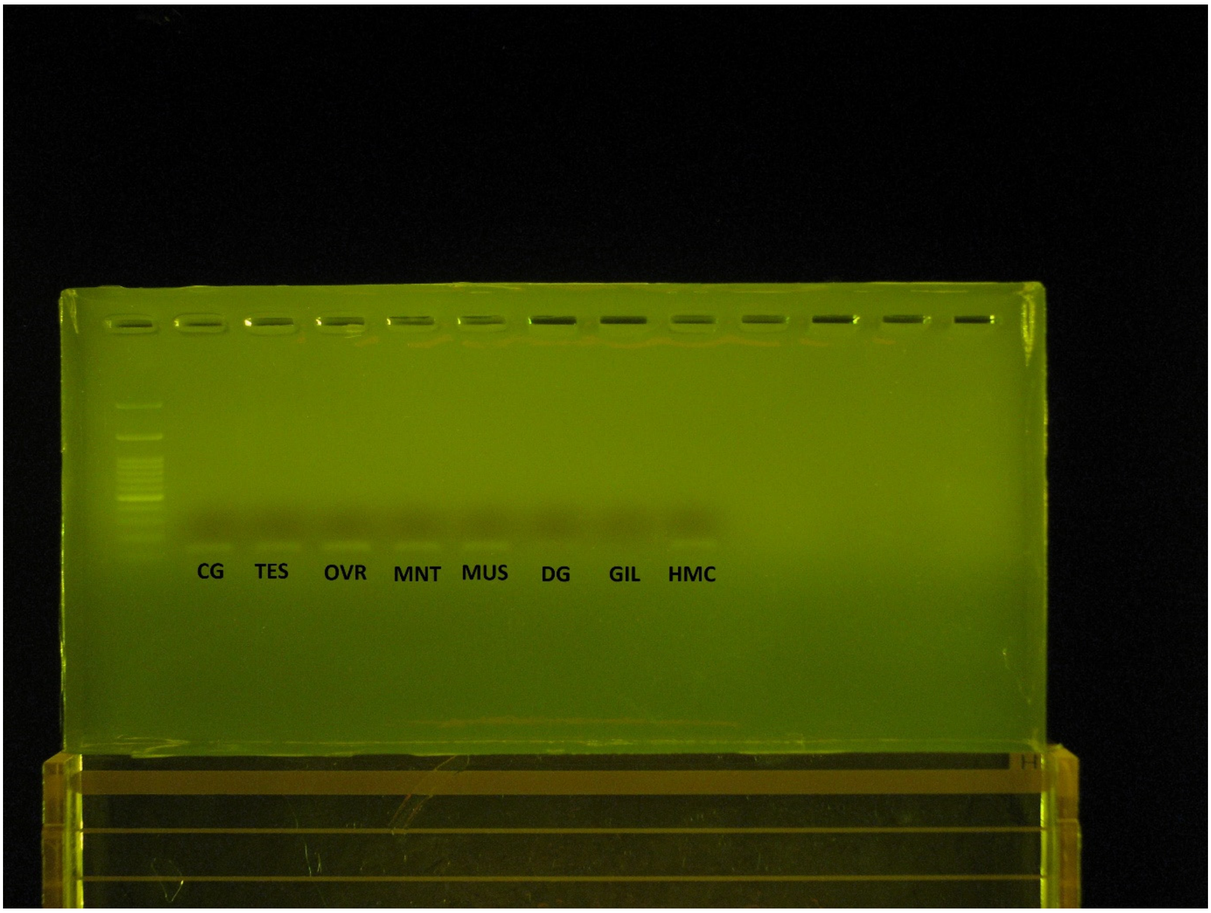

Figure S1: Tissue specific Semi-quantitative expression of *Hdh-SMP5* in Pacific abalone

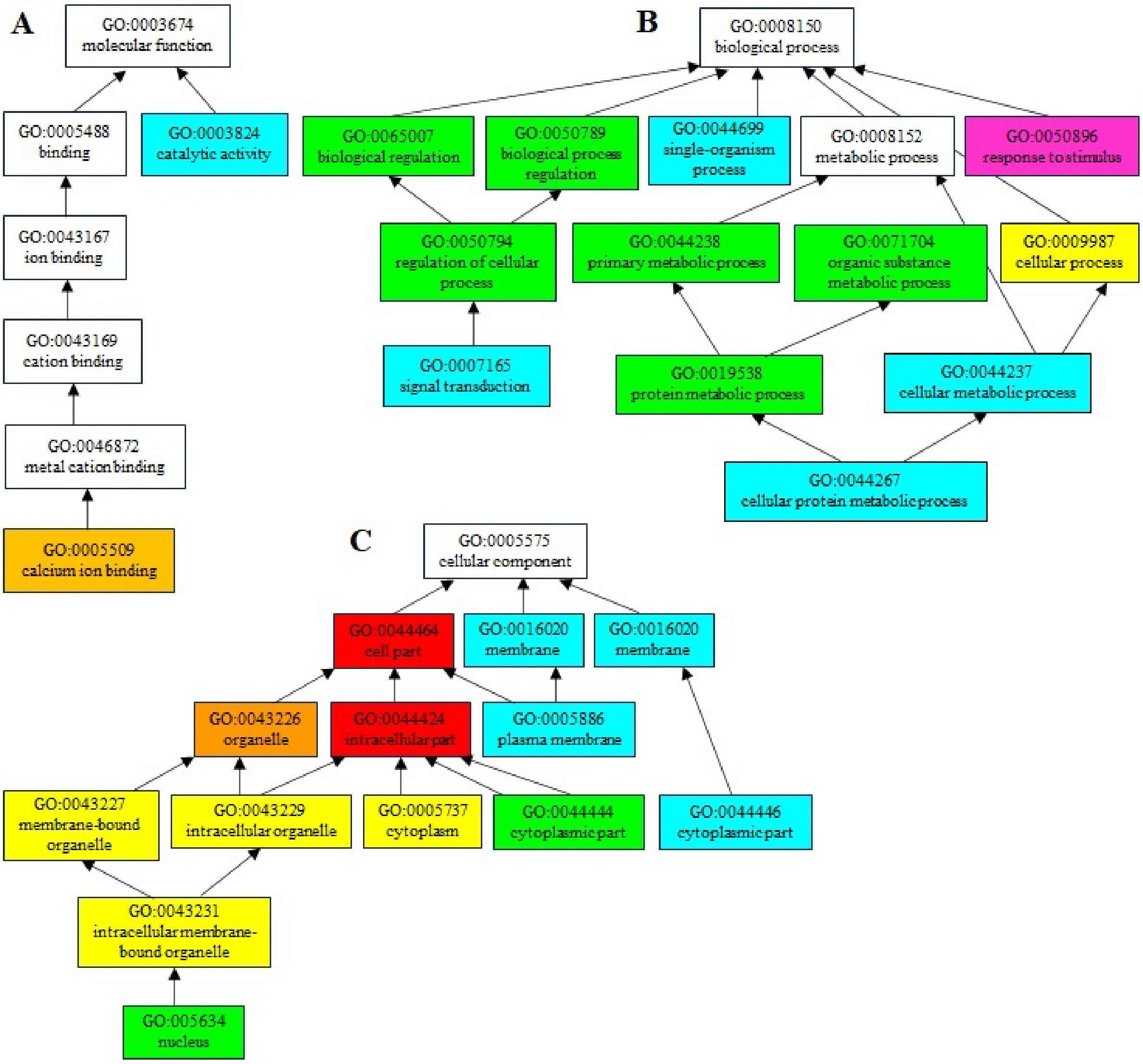

Figure S2: Function (gene ontology) analysis of *Hdh-SMP5* in Pacific abalone: S2A) molecular function, S2B) biological process, S2C) cellular component
